# Activation of Pedunculopontine Tegmental Nucleus Alleviates the Pain Induced by the Lesion of Midbrain Dopaminergic Neurons

**DOI:** 10.3390/ijms25115636

**Published:** 2024-05-22

**Authors:** Shiqiang Zhang, Jingjing Zhang, Yihao Yang, Weidong Zang, Jing Cao

**Affiliations:** 1 School of Basic Medical Sciences, Zhengzhou University, Zhengzhou 450001, China; zsq1989@gs.zzu.edu.cn (S.Z.); zhangjj0@mail.ustc.edu.cn (J.Z.); yangyh@gs.zzu.edu.cn (Y.Y.); 2 Neuroscience Research Institute, Zhengzhou University, Zhengzhou 450001, China

**Keywords:** pedunculopontine tegmental nucleus, Parkinson’s disease, dopamine, pain

## Abstract

The loss of midbrain dopaminergic (DA) neurons is the fundamental pathological feature of Parkinson’s disease (PD). PD causes chronic pain in two-thirds of patients. Recent studies showed that the activation of the pedunculopontine tegmental nucleus (PPTg) can effectively relieve inflammatory pain and neuropathic pain. The PPTg is located in the pontomesencephalic tegmentum, a target of deep brain stimulation (DBS) treatment in PD, and is involved in motor control and sensory integration. To test whether the lesion of midbrain DA neurons induced pain hypersensitivity, and whether the chemogenetic activation of the PPTg could modulate the pain, the AAV-hM3Dq receptor was transfected and expressed into the PPTg neurons of 6-hydroxydopamine-lesioned mice. In this study, von Frey, open field, and adhesive tape removal tests were used to assess animals’ pain sensitivity, locomotor activity, and sensorimotor function and somatosensory perception, respectively. Here, we found that the lesion of midbrain DA neurons induced a minor deficit in voluntary movement but did not affect sensorimotor function and somatosensory perception in the tape removal test. The results showed that lesion led to pain hypersensitivity, which could be alleviated both by levodopa and by the chemogenetic activation of the PPTg. Activating the PPTg may be a potential therapeutic strategy to relieve pain phenotypes in PD.

## 1. Introduction

Parkinson’s disease (PD) is a chronic progressive neurodegenerative disorder that primarily results in symptoms of motor dysfunctions, such as tremors, bradykinesia, and postural instability [1,2]. It is worth noting, however, that pain is also a common and distressing symptom of PD [3,4,5], afflicting 30 to 85% of PD patients [5,6,7]. Notably, the underlying mechanisms of pain or altered pain perception in PD are not fully understood, hindering its effective treatment. PD is characterized by the loss of midbrain dopaminergic (DA) neurons, indicating that a decrease in DA neurons disinhibits pain signals in PD patients [6,8,9]. However, the therapeutic efficacy of dopamine drugs in relieving pain symptoms associated with PD has been reported to be inconsistent [10,11], suggesting an urgent need to explore new treatment strategies. Sensory signals generated by primary nociceptors need to be sent to the spinal cord and eventually to the brain to create the sensation of pain. Such an anatomical basis leads to the conclusion that the generation of abnormal pain might be of central, peripheral, or concurrent origin, which makes pain management more difficult. Previous research showed that the inhibition of subthalamic nucleus (STN, a target of deep brain stimulation treatment in PD) neurons relieves somatic pain behaviors in Parkinsonian mice [12], and two recent studies showed that the activation of cholinergic neurons of the pedunculopontine tegmental nucleus (PPTg, another target of deep brain stimulation treatment in PD) can effectively relieve inflammatory pain and neuropathic pain [13,14]. The PPTg is involved in motor control and sensory integration [13,14,15,16,17], and could be a potential therapeutic target to relieve pain phenotypes in PD. Based on these considerations, we hypothesized that PD pain is mainly due to the loss of midbrain DA neurons. In this study, we established a mouse model of DA lesion-induced pain by unilaterally injecting 6-hydroxydopamine (6-OHDA) into the midbrain dopamine regions [9,18]. Subsequently, we tested whether the chemogenetic activation of the PPTg could ameliorate pain hypersensitivity in 6-OHDA-lesioned mice.

## 2. Results

### 2.1. Unilateral Lesion of Midbrain Dopaminergic Neurons Leads to Pain Hypersensitivity

**Figure 1:**
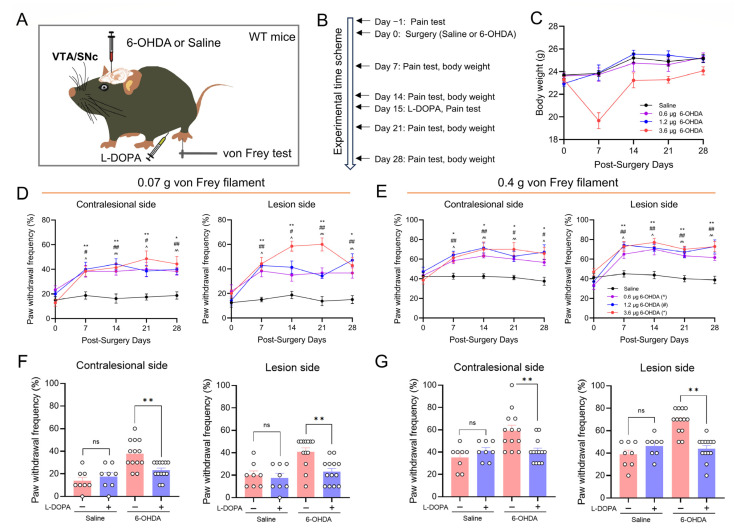
Unilateral 6-hydroxydopamine (6-OHDA)-lesioned mice exhibit mechanical pain hypersensitivity. (**A**) 6-OHDA lesion and L-DOPA application strategy. Mice received unilateral 6-OHDA injection targeting the ventral tegmental area (VTA) and substantia nigra pars compacta (SNc). (**B**) Experimental timeline. (**C**) Weight changes in mice following various doses of 6-OHDA injection (*n* = 6–8 per group). The paw withdrawal frequency (PWF) of these mice under mechanical stimulation of a 0.07 g (**D**) and 0.4 g (**E**) von Frey filament. Unilateral 6-OHDA-lesioned mice displayed bilateral mechanical pain hypersensitivity (*: Saline vs. 3.6 μg 6-OHDA; #: Saline vs. 1.2 μg 6-OHDA; ^: Saline vs. 0.6 μg 6-OHDA; */#/^: *p* < 0.05; **/##/^^: *p* < 0.01; *n* = 6–8 per group). The PWF of mice under mechanical stimulation of a 0.07 g (**F**) or 0.4 g (**G**) von Frey filament after L-DOPA intraperitoneal injection. L-DOPA treatment significantly reduced the PWF in bilateral hindpaws of 6-OHDA-lesioned mice (ns: *p* > 0.05 compared with pre-L-DOPA treatment in saline group; **: *p* < 0.01 compared with pre-L-DOPA treatment in 6-OHDA group; *n* = 8 for saline, *n* = 13 for 6-OHDA).

**Figure 2:**
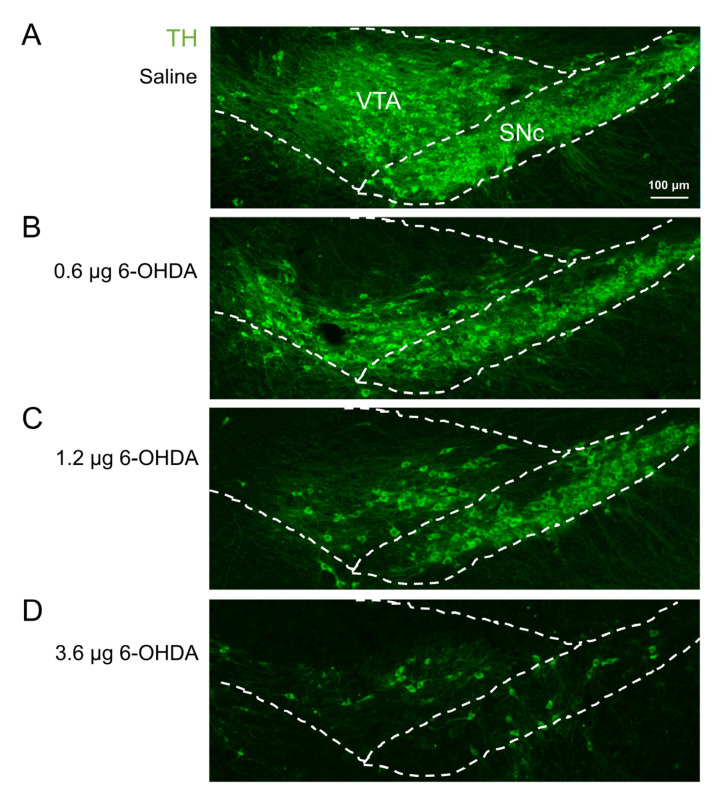
Lesion of DA neurons by 6-OHDA injection in the right VTA/SNc of mice. Following the behavioral tests, immunostaining for Tyrosine hydroxylase (TH) was performed (green). Images show the residual DA neurons in VTA/SNc after the injection of saline (**A**), 0.6 μg 6-OHDA (**B**), 1.2 μg 6-OHDA (**C**), and 3.6 μg 6-OHDA (**D**).

To simulate the DA neuron damage during PD, we injected various doses of 6-OHDA (0.6, 1.2, and 3.6 μg) into the ventral tegmental area (VTA) and substantia nigra pars compacta (SNc) of the right hemisphere of mice (Figure 1A,B). The mice injected with 3.6 μg 6-OHDA experienced significant body weight loss, especially at day 7 after the injection. Meanwhile, body weight was unaffected in the mice injected with 0.6 μg and 1.2 μg 6-OHDA (Figure 1C). The paw withdrawal frequency (PWF) to von Frey filaments at different time points after 6-OHDA injection was tested to show the change in pain sensitivity in 6-OHDA-lesioned mice (Figure 1B,D,E). When compared with mice receiving saline, mice injected with 6-OHDA had significantly higher PWF in bilateral hindpaws of mice between day 7 and day 28 post-injection. This indicated that the lesion of DA neurons in midbrain induced prominent persistent mechanical allodynia (Figure 1D,E). Following the behavioral tests, immunostaining for Tyrosine hydroxylase (TH) in VTA/SNc was performed (Figure 2). When compared with the saline group (Figure 2A), ~90%, ~70%, and ~50% VTA/SNc DA neuron loss was observed ipsilaterally in mice treated with 3.6 μg (Figure 2D), 1.2 μg (Figure 2C), and 0.6 μg 6-OHDA (Figure 2B), respectively.

**Figure 3:**
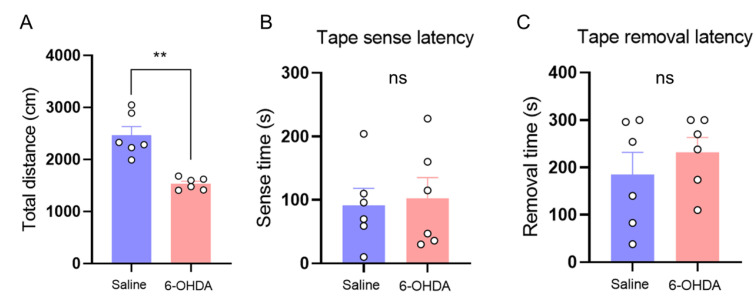
Lesion of midbrain DA neurons induced a minor deficit in voluntary movement but did not affect sensorimotor function and somatosensory perception in the tape removal test. (**A**) 14 days after 0.6 μg 6-OHDA injection, the mice exhibited about 30% deficit in the distance traveled in the open field test (**: *p* < 0.01; *n* = 6). After partial lesion of VTA/SNc DA neurons, mice showed no difference in the tape sense latency (**B**) (ns: *p* > 0.05; *n* = 6) or in the tape removal latency (**C**) (ns: *p* > 0.05; *n* = 6) when compared with the control mice.

In light of the effects of each dos of 6-OHDA on body weight, pain sensitivity, and DA lesion, we decided to used 0.6 μg 6-OHDA for all subsequent experiments so as to be in line with the 3Rs principles. An open field test revealed differences in voluntary movement between 6-OHDA-lesioned mice and saline-treated mice. On day 14 following 6-OHDA injection, the mice exhibited a ~30% deficit in the distance traveled (Figure 3A). Although 6-OHDA-lesioned mice showed a minor deficit in voluntary movement (Figure 3A), no group differences in sensorimotor function and somatosensory perception in the adhesive tape removal test were observed (Figure 3B,C).

To test whether the mechanical allodynia observed in 6-OHDA-lesioned mice was related to dopamine depletion, we injected levodopa (L-DOPA, 0.5 mg/kg, ip) to rescue dopaminergic neurotransmission and measured the PWF of mice 1 h later (Figure 1B). The result showed that L-DOPA treatment significantly reduced the PWF in bilateral hindpaws of 6-OHDA-lesioned mice, but had no effect in the saline-treated mice (Figure 1F,G).

These data indicate that the unilateral lesion of VTA/SNc DA neurons induces bilateral mechanical hypersensitivity, which may result from the dysfunction of dopaminergic neurotransmission in the brain.

### 2.2. Activation of the PPTg Alleviates the Mechanical Allodynia in 6-OHDA-Lesioned Mice

**Figure 4:**
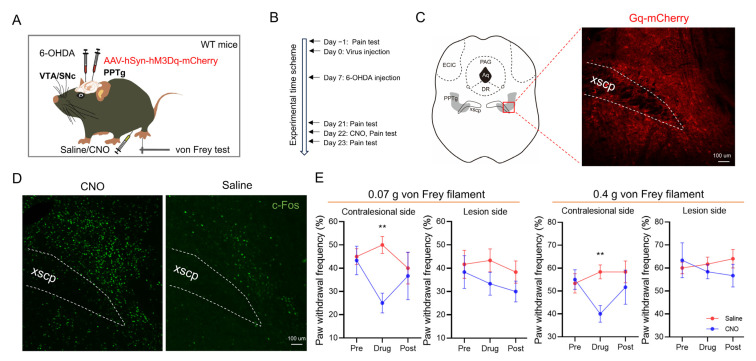
Pedunculopontine tegmental nucleus (PPTg) activation leads to pain relief in unilateral 6-hydroxydopamine (6-OHDA)-lesioned mice. (**A**) The chemogenetic activation strategy for the PPTg through hM3Dq (Gq) expression and clozapine-N-oxide (CNO) application with 6-OHDA-lesioned mice. (**B**) Experimental timeline. (**C**) PPTg chemogenetic targeting. (**D**) PPTg chemogenetic activation (c-Fos immunofluorescence, green). (**E**) The paw withdrawal frequency (PWF) of 6-OHDA-lesioned mice under mechanical stimulation of a 0.07 g or 0.4 g von Frey filament after CNO intraperitoneal injection. CNO treatment significantly reduced the PWF in the contralesional hindpaw (left hindpaw) of 6-OHDA-lesioned mice, but not the lesion-side hindpaw (**: *p* < 0.01; *n* = 6 per group).

To address whether the activation of the PPTg regulates pain sensitivity in 6-OHDA-lesioned mice, AAV (100 nL, AAV-hSyn-hM3Dq-mCherry) was infused directly into the right PPTg of 6-OHDA-lesioned mice (Figure 4A,B). The successful transfection of the virus in the PPTg was verified by the mCherry on frozen sections (Figure 4C). The degree to which PPTg neurons could be potentiated via chemogenetic activation was verified by c-Fos staining (Figure 4D). Clozapine N-oxide (CNO) treatment significantly reduced PWF in the contralesional side, but not the lesion-side, hindpaw of 6-OHDA-lesioned mice (Figure 4D). These results suggest that PPTg activation significantly alleviated midbrain DA lesion-induced mechanical allodynia.

## 3. Discussion

Our data indicate that the lesion of midbrain DA neurons leads to mechanical allodynia in mice, supporting the notion that dopamine depletion may induce physical pain [12,19]. We also show that the activation of the PPTg could alleviate mechanical allodynia in 6-OHDA-lesioned mice. To our knowledge, this is the first study to verify the relief of pain by chemogenetic modulation of the PPTg in a rodent 6-OHDA–lesion model. These findings may provide a neural substrate for pain management in PD.

Although our results show that the lesion of midbrain DA neurons can induce mechanical allodynia, the role of DA neurons in encoding pain has been controversial. Several studies have shown midbrain DA neurons to be inhibited by noxious stimuli [19,20], whilst others have demonstrated midbrain DA neurons to be excited by pain stimuli [21,22]. These discrepancies might have arisen due to the anatomical and functional heterogeneity of midbrain DA neurons [21]. The mechanisms underpinning dopamine dysfunction-induced pain are unclear. In the current study, we demonstrated that the acute lesion of midbrain DA neurons led to pain hypersensitivity, which could be reversed by L-DOPA.

Considering that a high dose of 6-OHDA induced remarkable body weight loss, we used a partial unilateral lesion model in this work to reduce the harm in mice. The dose of 0.6 μg 6-OHDA was microinjected into the VTA/SNc, resulting in a ~50% loss of VTA/SNc DA neurons. Whilst lesion was only partial, mild motor deficits were still observed in the open field test, but without affecting sensorimotor function and somatosensory perception in the adhesive tape removal test. Therefore, our model displayed good face validity to the symptoms of recently diagnosed PD patients. Whilst such patients are not impaired with regard to sensory-discriminative pain processing [6], they often exhibit pain phenotypes before motoric dysfunction becomes noticeable [23].

The PPTg, located in the dorsal pons, is composed of cholinergic, glutamatergic, and GABAergic neurons, which modulate locomotion [15], wakefulness [24], emotion [25], reward [26], and sensory integration [13]. In this work, we found that the activation of the PPTg could alleviate pain induced by 6-OHDA lesion in mice. Previous studies have shown the microinjection of glutamate into the PPTg produces a significant reduction in incisional pain in rats [27], and that the PPTg plays a crucial role in the antinociception induced by tonic and tonic–clonic seizures [28]. Recently, Sullere et al. identified a cholinergic circuit that relieves pain despite opioid tolerance [13]. They found that optogenetic activation of PPTg^ChAT+^→vlPAG (ventrolateral periaqueductal gray) projections could exert profound analgesia without altering motor function or anxiety-like behavior. Subsequently, Han et al. found that the stimulation of the PPTg^ChAT+^→SNr (substantia nigra pars reticulata) projection mitigates hyperalgesia in mice with acute and chronic pain [14]. These reported analgesic effects of the PPTg are consistent with our finding in 6-OHDA-lesioned mice, which strongly supports the notion that the PPTg is involved in the regulation of pain. The output regions of the PPTg include subthalamic nucleus, VTA, SNc/SNr, globus pallidus, ventral posterior complex of the thalamus, lateral hypothalamus, and amygdaloid nuclear complex [29,30]. Neither how these circuits normally function nor their role in pain have been well studied and, thus, require more research, and that is what we are doing next. Above all, however, activating the PPTg appears to be a potential therapeutic strategy for pain hypersensitivity phenotypes in PD.

## 4. Materials and Methods

### 4.1. Animals

Adult male C57BL/6 mice (8 weeks old) were purchased from HuaFuKang (Beijing, China) Biotechnology Co., Ltd. All mice were group-housed (5 per cage) in a temperature- and humidity-controlled environment on a 12 h light/dark cycle, with free access to water and food. The use of the animals followed the guidelines of the International Association for the Study of Pain, and was approved by the Animal Care and Use Committee of Zhengzhou University (No.: ZZUIRB G2R 2019-0344; Approved date: 2020.01–2023.12).

### 4.2. Surgery

For all surgeries, mice were anesthetized with isoflurane (2–3% for induction, 1–2% for maintenance, RWD Life Science, Shenzhen, China), shaved using a trimmer, and head-fixed on the stereotaxic apparatus (Stoelting, Inc., Wood Dale, IL, USA). Stereotaxic injections were performed as previously described [13]. Body temperature was maintained at 37 °C using a homeothermic heating pad. After drug or virus injection, the needle was held in place for 10 min to ensure adequate viral diffusion, and then slowly withdrawn.

*Dopaminergic (DA) neuron lesion*: Mice received desipramine hydrochloride injection (25 mg/kg, i.p., GlpBio, Montclair, CA, USA) half an hour before anesthesia. When anesthetized by isoflurane, mice were fixed to the stereotaxic apparatus and injected with 0.6, 1.2, or 3.6 μg 6-hydroxydopamine (6-OHDA, 12 µg/µL in 0.2% ascorbic acid, GlpBio, Montclair, CA, USA) or 0.9% saline into the right VTA/SNc according to the following coordinates: anteroposterior (A/P) = −3.15 mm, mediolateral (M/L) = −0.55 mm, dorsoventral (D/V) = −4.50 mm (*Paxinos and Franklin’s The Mouse Brain in Stereotaxic Coordinates*, 4th Edition).

*AAV injection*: For chemogenetic manipulations, the virus (AAV-hSyn-hM3Dq-mCherry, 5 × 10^12^ vg/mL, Brain VTA, Wuhan, China) was injected in a volume of 100 nL at a flow rate of 10 nL/min in the right pedunculopontine tegmental nucleus (PPTg, A/P: −4.65 mm, M/L: −1.25mm, D/V: −3.50mm). A 3-week interval was set between the virus injection and the behavioral tests to ensure the expression of virus.

### 4.3. L-DOPA and CNO Treatment

To increase the dopamine in the brain, mice were administered with levodopa (L-DOPA, 0.5 mg/kg, i.p., Sigma-Aldrich, Saint Louis, MO, USA) 1 h prior to the pain behavioral test [12]. For chemogenetic manipulations, mice were administered with clozapine-N-oxide (CNO, 0.3 mg/kg, i.p., Brain VTA, Wuhan, China) 1 h prior to the pain behavioral test [24].

### 4.4. Behavioral Tests

All the behavioral tests were conducted in the light period of the light/dark cycle under temperature- and humidity-controlled conditions. Before the first behavioral tests, mice were put into the test chamber for 2 times (1 h/d) to acclimate to the test chamber and room. All the behavioral tests were conducted in a double-blind manner.

*von Frey filament test*: As previously described, mechanical sensitivity of the hindpaw was tested with manual repeated applications of 0.07 g and 0.4 g von Frey filaments (Stoelting, Inc., Wood Dale, IL, USA) to the central plantar [31]. Each hindpaw was measured 10 times for each filament. The inter-stimulus interval was ≥5 s during the 10 repeats of the stimulation to one plantar. When changing the filament strength or the test plantar, the interval time should be ≥10 min. The paw withdrawal frequency to the 10 repeats of one filament was recorded and analyzed.

*Open field test*: Locomotion function was examined via open field test [32]. Mice were placed into a gray Plexiglas box (45 × 45 × 45 cm^3^) to freely explore and recorded for 5 min by a computer camera. Distance traveled during the test was analyzed using the video tracking system of Smart v3.0 software (Panlab Harvard Apparatus, Cornellà, Barcelona, Spain). After each test, the open field arena was cleaned with 75% ethanol.

*Adhesive tape removal test*: Sensorimotor function and somatosensory perception were examined via adhesive tape removal test. Mice were habituated for at least 30 min in a clean cage. As previously described, a small adhesive tape strip (0.4 cm × 0.4 cm) was attached to the plantar surface of the left hindpaw [33]. After a latency period, the mouse tried to remove the tape. The latencies for mice to contact the tape (sense latency) and to remove the tape (removal latency) were recorded. Mice were trained by performing 1 trial per day for 4 d before testing. After each test, the cage was cleaned with 75% ethanol.

### 4.5. Immunohistochemistry

Mice were deeply anesthetized with isoflurane and perfused with saline, followed by 4% paraformaldehyde (PFA). The brains were extracted and soaked in 4% PFA at 4 °C overnight, and then gradient dehydrated with 20% and 30% sucrose solution, respectively. Coronal brain sections (35 μm) were cut using a freezing microtome (CM1950, Leica Biosystems, Wetzlar, Germany). For immunostaining, brain slices were sequentially washed in 0.01 M phosphate-buffered saline (PBS, 10 min each time, 3 times), blocked in 10% goat serum and 0.3%Triton in PBS for 2 h at room temperature, incubated with primary antibody (rabbit anti-c-Fos, 1:1000, Abcam, Boston, MA, USA; rabbit anti-TH, 1:3000, ImmunoStar, Hudson, WI, USA) in 5% goat serum and 0.3% Triton in PBS at 4 °C overnight, washed 3 times (10 min each) in PBS, incubated with secondary antibody (Alexa Fluor 488–conjugated goat anti-rabbit, 1:300, Jackson ImmunoResearch, West Grove, PA, USA) at 37 °C for 2 h, washed 3 times (10 min each) in PBS, moved onto glass slides, dried in the dark, and then cover-slipped in antifade mounting medium. The images were captured using under a Nikon Ni-U fluorescence microscope (Nikon, Tokyo, Japan). The brains of mice injected with virus were extracted 1 h after CNO or saline was given.

### 4.6. Statistical Analysis

Data are expressed as mean ± standard error of mean (SEM) and analyzed with GraphPad Prism 8 (GraphPad Software, lnc., La Jolla, CA, USA). Each dataset was tested for normality of distribution prior to analysis. For two-group analysis, an unpaired Student’s *t*-test was used. For the analysis among multiple groups, a one-way or two-way ANOVA followed by Tukey’s multiple comparisons test was applied. *p* < 0.05 was considered statistically significant.

## 5. Conclusions

Based on our findings, we conclude that the lesion of midbrain DA neurons induces pain hypersensitivity, which can be relieved by chemogenetic activation of the PPTg. This result indicates a new potential therapeutic strategy for pain phenotypes in PD.

## Data Availability

Raw data are available upon reasonable request.
